# Cumulative Effects of Strontium Ranelate and Impact Exercise on Bone Mass in Ovariectomized Rats

**DOI:** 10.3390/ijms22063040

**Published:** 2021-03-16

**Authors:** Priscilla Aveline, Annabelle Cesaro, Marija Mazor, Thomas M. Best, Eric Lespessailles, Hechmi Toumi

**Affiliations:** 1 I3MTO, Université d’Orléans, 45000 Orléans, France; priscilla.aveline87@gmail.com (P.A.); annabelle.cesaro@univ-orleans.fr (A.C.); eric.lespessailles@chr-orleans.fr (E.L.); 2 Center for Proteomics University of Rijeka, Faculty of Medicine Branchetta, 51000 Rijeka, Croatia; mazor.marija@gmail.com; 3 UHealth Sports Medicine Institute, Department of Orthopedics, Division of Sports Medicine, University of Miami, Miami, FL 33136, USA; txb440@med.miami.edu; 4 Département de Rhumatologie, Centre Hospitalier d’Orleans, 45100 Orléans, France; 5 Plateforme Recherche Innovation Médicale Mutualisée d’Orléans, Centre Hospitalier d’Orleans, CEDEX 02, 45067 Orleans, France

**Keywords:** strontium ranelate, impact exercise, ovariectomized rats, bone status

## Abstract

OBJECTIVE: To explore the effect of physical exercise (EXE), strontium ranelate (SR), or their combination on bone status in ovariectomized (OVX) rats. DESIGN: Sixty female Wistar rats were randomized to one of five groups: sham (Sh), OVX (O), OVX+EXE (OE), OVX+SR (OSR), and OVX+EXE+SR (OESR). Animals in EXE groups were subjected to 10 drops per day (45 cm in height); rats in SR groups received 625 mg/kg/day of SR, 5 days/week for 8 weeks. Bone mineral density (BMD) and bone mineral content (BMC, dual-energy X-ray absorptiometry (DXA)), mechanical strength of the left femur (three-point bending test), and femur microarchitecture of (micro-computed tomography imaging, microCT) analyses were performed to characterize biomechanical and trabecular/cortical structure. Bone remodeling, osteocyte apoptosis, and lipid content were evaluated by ELISA and immunofluorescence tests. RESULTS: In OVX rats, whole-body BMD, trabecular parameters, and osteocalcin (OCN) levels decreased, while weight, lean/fat mass, osteocyte apoptosis, and lipid content all increased. EXE after ovariectomy improved BMD and BMC, trabecular parameters, cross-sectional area (CSA), moment of inertia, and OCN levels while decreasing osteocyte apoptosis and lipid content. SR treatment increased BMD and BMC, trabecular parameters, CSA, stiffness, OCN, and alkaline phosphatase (ALP) levels. Furthermore, fat mass, N-telopeptide (NTX) level, osteocyte apoptosis, and lipid content significantly decreased. The combination of both EXE and SR improved bone parameters compared with EXE or SR alone. CONCLUSION: EXE and SR had positive and synergistic effects on bone formation and resorption.

## 1. Introduction

Treatment strategies in osteoporosis are based on both pharmacological [1] and non-pharmacological approaches to treatment, including fall prevention, calcium and vitamin D supplementation, exclusion of tobacco, and physical exercise (EXE) [2,3].

Pharmacologic treatments for osteoporosis generally target either bone formation or bone resorption [4,5]. However, one agent, strontium ranelate (SR), acts on both bone formation and resorption [6,7,8,9]. Previous studies have shown that SR administration significantly reduced the risk of vertebral, non-vertebral, and hip fractures in postmenopausal women [4]. SR administration reduces bone resorption and stimulates bone formation [10] by increasing osteoblast differentiation, decreasing osteoclast differentiation, and increasing synthesis of collagen and non-collagen proteins [10,11,12]. SR induces a decrease in receptor activator of nuclear factor kappa-B ligand (RANKL) and an increase in osteoprotegerin (OPG) concentrations, leading to a modification of the RANKL/OPG ratio in favor of osteoblast differentiation [8,11,13]. It is interesting to note that SR has been observed to have no true effect on the intracellular RANKL protein, but to have an indirect influence via the significant decrease in the membranous localization of RANKL [14].

In 2007, Chattopadhyay et al. showed that SR activates osteoblasts through the calcium-sensing receptor (CaSR) [6]. The interaction of SR with CaSR induced the activation of mitogen-activated protein kinase signaling, thereby increasing osteoblast replication [6,15]. In 2010, Takao et al. demonstrated that SR induces not only osteoblast proliferation but also its differentiation and mineralization by activating the CaSR [16]. Moreover, they confirmed that the therapeutic efficacy of SR for osteoporosis may be partly mediated by the CaSR [16]. CaSR activation not only acts on osteoblasts but also leads to osteoclast apoptosis [17]. However, SR does not only act via the CaSR. In 2009, Fromigue et al. showed, using CaSR knockout mice, that SR promotes osteoblast replication independently of the CaSR/extracellular signal-regulated kinases (Erk1)/2 cascade [15]. These results confirm two different pathways for SR to modulate osteoblasts.

In parallel, several studies have highlighted that some EXE protocols can increase bone density and bone mass in both animals [18,19] and humans [20,21,22]. This mechanical stress modulates bone cell metabolism by osteocyte stimulation through some mechanosensitive molecules such as piezo1 or by the key differentially expressed genes (DEGs) [23,24,25]. Previous studies showed that osteocytes were found to be the most responsive cell type to interstitial fluid flow in vitro [26], and changes in osteocyte morphology and the direction of loadings affect the cell stimulation [27]. Various factors monitor the interstitial fluid flow including loading, frequency, stress distribution, vibration, and recovery phase [27,28]. All these elements can influence the bone response to exercise.

Previous studies have explored the effects of combined anti-osteoporotic treatment with EXE on bone health with variable results [29,30,31]. For example, a combination of EXE (treadmill exercise: 5% incline, 22–24 m/min, 60 min/day, 5 days/week for 14 weeks) combined with alendronate was more beneficial in preventing declines in bone mass and strength [29] than anti-osteoporotic treatment alone. Another study using a bisphosphonate (risedronate) combined with EXE (swimming) showed better effects on bone mass preservation than either treatment alone [32]. However, Lespessailles et al. reported that EXE (treadmill exercise: 15 m/min, 60 min/day, 5 days/week for 12 weeks) or zoledronate alone prevented bone loss and ameliorated bone strength while the combination did not produce any synergetic or additive effects [30]. In 2015, we showed that combination of EXE (treadmill interval training exercise 1 h/day, 5 days/week for 9 weeks) and sclerostin (SOST) antibody treatment in glucocorticoid-treated rats did not show additional improvement in bone mass or biomechanical parameters compared with either treatment alone [33]. Herein, we evaluated whether bone EXE and SR treatment together would have synergistic or additive effects on bone status in an ovariectomized rat model.

## 2. Results

### 2.1. Effects of Ovariectomy on Bone

Ovariectomy led to an alteration of several bone parameters. In the O group, whole-body BMD decreased significantly (−25.64%) compared to the Sh group (Table 1). We also observed a significant increase in the weight (+43.48%) and the fat and lean mass (+31.66% and +57.98%, respectively) in the O group compared to the Sh group (Table 1).

Regarding trabecular and cortical bone parameters, the O group showed a significant decrease in BV/TV (−71.43%), Tb.Th (−10.68%), Tb.N (−68.13%), Ct.Th (−13.05%), and Po.N (−6.36%) compared to Sh (Table 2), while CSA increased (+15.52%) (Table 3).

**Figure 1:**
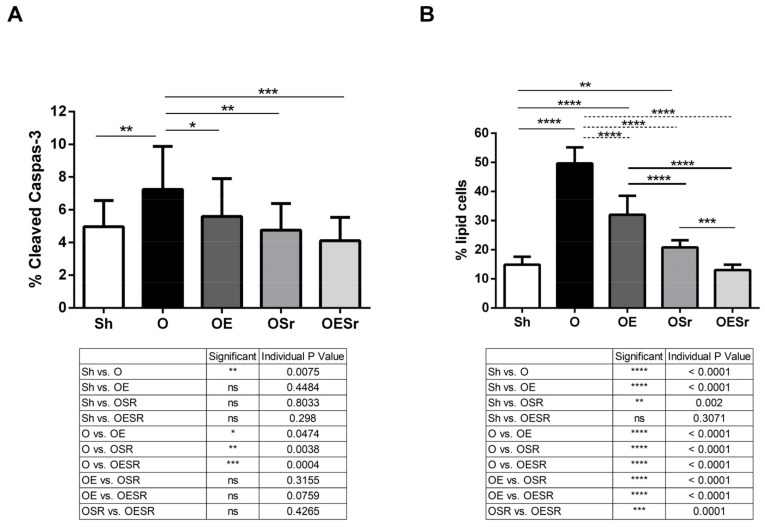
Cortical bone staining. (**A**) Osteocyte apoptosis represented by % of cleaved caspase-3 immunostaining. (**B**) Percent of lipid cell staining by Nile red. Rats in the O group were ovariectomized. Rats in OE and OESr were subjected to 10 impacts per day, 5 days a week for 8 weeks. Rats in Sr and OESr groups received 625 mg/kg/day of SR. The critical *p*-value was * *p* ≤ 0.05, ** *p* ≤ 0.005, *** *p* ≤ 0.0005, **** *p* ≤ 0.0001; ns: non-significant.

For cell bone metabolism, we observed a decrease in ALP in the O group (−21.52%, Table 4) and an increase in lipid content (+233.87%) and osteocyte apoptosis (+46.04%) compared to the Sh group (Figure 1A).

### 2.2. BMD, BMC, and Body Composition

Whole-body BMC and BMD significantly increased in the OE (+46.48% and +48.28%, respectively), OSr (+48.59% and +51.72%, respectively), and OESr (+58.10% and + 62.07%, respectively) groups compared to the O group (Table 1). However for BMC and BMD, no significant difference was observed between the EXE, SR, or EXE + SR groups.

Body weight (BW) decreased significantly for the OESr group compared to the other groups (−43.71% versus O, −36.45% versus OE, and −31.63% versus OSr) (Table 1). Furthermore, fat mass decreased significantly in the OESr group compared to the O (−64.86%), OE (−59.87%), and OSr (−54.63%) groups.

### 2.3. Trabecular Bone Microarchitecture

We investigated the effects of EXE, SR, or EXE+SR on trabecular bone microarchitecture (BV/TV, Tb.Th, and Tb.N). BV/TV increased in the OE (+202.48%), OSr (+253.54%), and OESr groups (+324.29%) compared to the O group (Table 2). Tb.Th and Tb.N were increase in the OE group (+34.78% and +122.84%, respectively), in the OSr group (+53.26% and +130.89%, respectively), and in OESr (+67.39% and +154.61%, respectively) compared to the O group. The Fisher LSD test showed a synergistic effect of EXE+SR for BV/TV and Tb.N parameters.

### 2.4. Cortical Bone Microarchitecture

Ct.Po and Po.N decreased significantly in the OESr group (−61.54% and −57.14%, respectively) while Ct.Th increased significantly in the SR (+21.79%) and OESr (+23.97%) groups. The Fisher LSD test showed a synergistic effect of EXE+SR for Ct.Po and Po.N (Table 2).

### 2.5. Biomechanics

Ultimate strength increased significantly in the OESr group (+9.24%) compared to the O group. For cross-sectional area (CSA), the OE, OSr, and OESr groups showed a significant increase compared to the O group (+9.66%, +8.45%, and +15.76%, respectively). For moment of inertia, only the OE and OESr groups showed a significant increase compared to the O group (+15.30% and +19.88%, respectively) (Table 3).

For stiffness, a significant increase was observed in the OSr (+8.07%) and OESr groups (+14.98%) compared to the O group. The Fisher LSD test showed a synergistic effect of EXE+SR for stiffness (Table 3).

For yield point stress, no significant differences were found for any of the conditions (Table 3).

### 2.6. Bone Turnover

ALP concentration increased significantly for the OSr (4.189 ± 0.839 U/L) and OESr groups (3.110 ± 0.731 U/L) compared to the O group (2.314 ± 0.844 U/L), while the OE group showed no difference (Table 4). For OCN concentration, all groups, including the OE (1.538 ± 0.364 U/L), OSr (1.658 ± 0.466 U/L), and OESr (1.879 ± 0.698 U/L) groups, showed a significant increase compared to the O group (1.222 ± 0.346 U/L). The Fisher LSD test showed a synergistic effect of EXE and SR on OCN concentration, a bone formation marker (Table 4).

For the bone resorption marker NTX, we observed a significant decrease in the OSr (0.585 ± 0.086 U/L) and OESr (0.622 ± 0.083 U/L) groups compared to the O group (0.857 ± 0.076 U/L) (Table 4).

### 2.7. Osteocyte Apoptosis and Lipid Cells

We observed a decrease in osteocyte apoptosis for the OE, OSr, and OESr groups compared to the O group (Figure 1A).

There were significantly fewer cells stained with Nile red in the OE, OSr, and OESr groups compared to the O group (Figure 1B). Furthermore, we observed that the OSr and OESr groups showed a significantly lower number of positive cells compared to the OE group (Figure 1B).

## 3. Discussion

There is limited knowledge in postmenopausal women and animal models mimicking osteoporosis on the potential associated effects of pharmaceutical activity and anti-osteoporotic pharmaceutical agents on bone health [8,9,10]. Herein, to advance further osteoporosis management, we have explored the synergic or additive effect of SR administration and EXE in the ovariectomized rat model. Our results showed that both SR and impact exercise had beneficial effects on bone tissue in enhancing bone microarchitecture and strength. Additionally, we found the combined intervention of EXE and SR to be superior in decreasing weight, fat mass, Ct.Po, and Po.N and increasing trabecular parameters, stiffness, and OCN level. In the present study, we provided a new insight into the impact of the combination of both treatments, SR and EXE, on bone status and metabolism.

Consistent with previous studies [11,12], ovariectomy in young female rats resulted in higher weight body gain (+43.5%), fat, and lean mass (+58% and +31.7%, respectively) compared to the Sh group, despite receiving a similar quantity of food. In 1987, McElroy et al. showed that ovariectomy in rats induces rapid weight gain in rats only 5 weeks after the ovariectomy procedure [13]. Furthermore, we observed that ovariectomy also had a significantly deleterious effect on whole-body BMC (−25.6%). These negative effects on bone composition and BMC were associated with deleterious effects on trabecular bone microarchitecture parameters. Our results showed a decrease in bone microarchitecture for trabecular and cortical parameters. Trabecular bone loss was also associated with significant modifications in the bone turnover marker osteocalcin [11]. In the present study, the ovariectomy induced osteocyte apoptosis, as previously described [14], and a lipid increase in bone cortical tissue compared to sham rats.

Ovariectomy in rats resulted in a body weight gain. Interestingly, we observed that the OE group had a higher body weight gain compared to the Sh group. These results indicate that EXE alone is not efficient to counterbalance the effect of OVX on weight. For SR treatment and both treatments (SR+EXE), we observed that the OSr group showed similar body weight gain and the OESr group showed lower body weight gain compared to the Sh group. SR treatment alone and the combination of SR and EXE suppress the OVX effects on body weight with a synergic effect of both treatments. Our protocol confirmed previous findings [13] and showed that fat mass increased after ovariectomy. Herein, SR treatment prevents these increases, and the combination of SR and EXE leads to a strong decrease in fat mass. In all groups, a higher lean mass was observed compared to sham rats. Interestingly, in the OESr group, the lean mass percent was higher compared to all groups. This result is in line with those obtained by Komrakova et al. in 2016 [15]. They observed that whole-body vibration could be applied in combination with SR treatment to improve muscle tissue [15]. However, the direct effects of SR on muscle tissue are still unknown.

Ovariectomy leads to a decrease in whole-body BMC and BMD [16]. EXE, SR, and combination of EXE and SR counterbalanced the OVX effects on BMC and BMD. Honda et al. in 2003 showed a similar effect of EXE on BMD [16]. Concerning SR, Ammann et al. in 2004 showed that SR treatment increased BMC and BMD in intact female rats [1]. The improved BMC and BMD obtained in the OESr group were similar to those obtained in the OE and OSr groups, confirming no synergic or additive effect.

Microarchitecture analysis showed that ovariectomy had negative effects on trabecular and cortical bone microarchitecture: BV/TV, Tb.Th, and Tb.N. We observed that EXE improves BV/TV; however, this parameter remains lower than the BV/TV of the Sh group. On Ct.Po and Ct.Th, EXE had no effect. This is in line with a previous study showing no changes in cortical bone parameters after physical exercise [16]. Furthermore, we also found no significant effect on biomechanical parameters after EXE. We observed that SR treatment suppresses the deleterious effect of ovariectomy on BV/TV and both cortical bone parameters. These results are similar to those obtained by Ammann et al. in 2004 with SR treatment on bone parameters in the OVX rat model [1]. Note, also, that the biomechanical properties were also improved by SR treatment. These results are consistent with the correlation already established between the mechanical properties of bone and cortical bone parameter changes [17]. The combination of both treatments, SR and EXE, improved the mechanical properties of bone and cortical bone parameters more than EXE or SR alone. Furthermore, statistical analysis showed that the combined intervention of EXE and SR had a synergic effect on BV/TV, Tb.Th, Tb.N, Ct.Po, and Po.N parameters and, thus, on the stiffness, the ultimate strength, the cross-sectional area, and the moment of inertia of bone.

Concerning bone turnover markers, EXE led to an increase in OCN level and a decrease in NTX level. This may suggest an advantage for bone formation. A previous study showed that EXE after ovariectomy increased OCN and ALP levels and decreased NTX level [16]. Klein-Nulend et al. in 2005 and then in 2012, suggested that changes in OCN, ALP, and NTX levels are a consequence of mechanical stress induced by EXE [18,19]. Before these studies, Umemura et al. demonstrated, in 1995, that jump training after ovariectomy was efficient in bone regeneration [2]. Surprisingly, we observed that EXE did not decrease osteocyte apoptosis and lipid number in cortical bone. For the SR-treated group, bone turnover markers were also modified in favor of bone formation: increases in ALP and OCN and a decrease in NTX. Furthermore, in this group, and in line with previous reports [20,21], osteocyte apoptosis and lipid number in cortical bone were significantly reduced. Interestingly, a combination of SR and EXE increased OCN levels with a synergic effect. However, although ALP increased and NTX level decreased, no synergic effect was noted. Osteocyte apoptosis and lipids in cortical bone were significantly decreased in SR+EXE compared to OVX. Furthermore, we observed a higher decrease in this group compared to the SR group. Surprisingly, only SR treatment decreased osteocyte apoptosis and lipids in cortical bone while EXE had no impact. This may indicate an additive effect of EXE to the SR treatment.

The present study is the first showing a synergic effect of SR and EXE treatment in ovariectomized rats. This is important as it leaves open the possibility that SR and EXE may act through different signaling pathways in bone. The signaling pathways involved in SR response in bone are mostly calcium-sensing receptor (CaSR)-dependent [22]. Previous studies showed that SR, through CaSR, stimulates osteoblast differentiation and proliferation and osteoclast apoptosis [20,21,22]. SR, by activation of CaSR signaling pathways, induces a decrease in RANKL and an increase in OPG. These changes in RANKL and OPG levels lead to a modification of the RANKL/OPG ratio in favor of bone formation [20,21,22]. In 2010, Fromigue et al. investigated SR’s effects on calcineurin (Cn)/nuclear factor of activated T cells (NFAT) signaling in osteoblast [23]. Wingless-related integration site (Wnt) proteins are known to play an important role in the control of cell replication, differentiation, and survival. In conclusion, SR, through CaSR pathways, Cn/NFATc1 pathways, and Wnt signaling, leads to an increase in bone formation, bone density, and bone strength.

Several studies highlighted that EXE protocols increase bone density and bone mass in animal models [24,25] and humans [9,26,27]. These bone modifications are due to osteocyte biological modification, which is the most responsive cell type, compared to osteoblasts, to interstitial fluid flow [28,29]. Parameters influencing bone remodeling by interstitial fluid flow responses are loading, frequency, stress distribution, vibration, and recovery phase [29,30,31]. Deformation of the bone matrix caused by mechanical loads is detected by osteocytes, which, in turn, send paracrine signals to osteoblasts and osteoclasts. As already mentioned, Wnt/lipoprotein receptor-related protein (LRP5) pathways have an important role in bone cell differentiation, proliferation, and apoptosis [30,31]. Various publications show that the Wnt/β-catenin signaling pathway is required for bone formation in response to mechanical loading [31,32]. Wnt/LRP5 pathways can be regulated by a SOST protein, the protein product of the Sost gene [33]. This protein is, therefore, a potent inhibitor of bone formation. Interestingly, a publication shows that mechanical loading reduces SOST levels in bone, suggesting that osteocytes might coordinate the osteogenic response to mechanical force by locally unleashing Wnt signaling [34]. However, mechanotransduction is complex, and diverse molecular cascades activated by mechanical stimuli are involved in cell bone response to physical exercise [19]. Various studies using a murine model with ubiquitous or osteoblast/osteocyte-specific deletion of genes highlight altered responses to mechanical loading in different signaling pathways, such as LRP5 [32], purinergic receptor P2X7 [35], and connexin 43 [36]. In these models, the defective response to loading could be due to a failure of the osteocytes to detect mechanical stimuli or to transmit signals to the other bone cells.

Taking into account the SR and EXE data, we hypothesize that in our model, the synergic effect of SR and EXE observed could be due to a modification of RANKL and OPG levels in favor of bone formation and activation of Wnt signaling by SR and EXE and stimulation of other signaling pathways (MAP kinases, P2X7) by EXE. Therefore, a combination of SR and EXE resulted in biological modifications in osteoblast and in osteocyte leading to an increase in the stiffness, ultimate strength, cross-sectional area, and moment of inertia of bone. Our study demonstrated that SR, EXE, and the combination of both prevented bone degradation after ovariectomy in the rat model. SR showed more positive effects on both bone resorption and formation compared to EXE. Yet the best improvements were noted in the combined group (SR and EXE) and notably on bone remodeling. These results may open a new therapeutic strategy in the management of osteoporosis and, in particular, for the treatment of postmenopausal osteoporosis.

Limitations: Note that recently, some adverse reactions have been reported resulting from the use of SR. This includes skin reactions and cardiovascular disorders including venous thromboembolism and myocardial infarction. Consequently, restrictions were made by the European Medicines Agency (European Medicines Agency) In the present stud, the treatment period was short, which may explain the absence of adverse reactions. Note, however, that the animal cardiovascular system was not examined.

## 4. Materials and Methods

### 4.1. Animals and In Vivo Experimental Design

**Figure 2:**
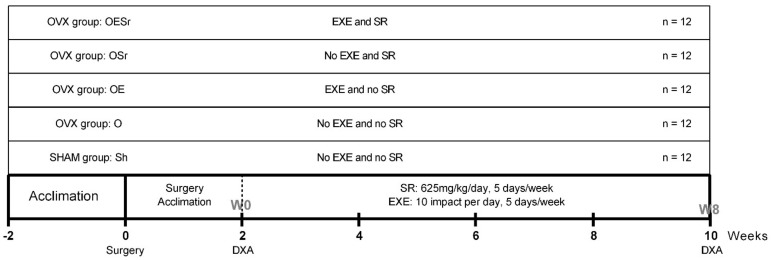
Study design. After acclimation, 60 female Wistar rats were randomly assigned to one of 5 groups (*n* = 12): SHAM (Sh), OVX (O), OVX+EXE (OE), OVX+SR (OSr), and OVX+EXE+SR (OESr). Note: OVX—ovariectomized; EXE—exercise; SR—strontium ranelate.

All experiments were approved by the animal protection committee of the University of Orleans, France (agreement n°: CEEA VdL; delivered: 2011-11-2). Sixty female Wistar rats, aged 5 weeks old at the beginning of the experiment, were purchased from Janvier Labs (Janvier Animal Production, Le Genet-St-Isle, France). Animals were housed two per cage with ad libitum access to water and feed (M2,SDS, France). They were maintained in a controlled temperature (22 ± 3 °C) environment and under 12/12 h light–dark cycles for the duration of the experiment. After one week of acclimation, the rats were randomly assigned (randomization and blinding method) to one of 5 groups: control/sham (Sh), ovariectomy (O), ovariectomy + EXE (OE), ovariectomy + SR treatment (OSr), and ovariectomy + EXE + SR treatment (OESr) (Figure 2). As noted in Figure 2, there were 12 animals per group.

For ovariectomies and sham operations, the rats were anesthetized with a mixture of ketamine–xylazine (80–10 mg/kg, intraperitoneally, Panpharma, Paris, France) and received intraperitoneal injection of meloxicam (2 mg/kg, Metacam, Paris, France) and an injection of buprenorphine (0.05 mg/kg, Burprecare, Paris, France) to prevent infection and pain. All rats were weighed once a week. At the end of experiments, all animals were euthanized with an intraperitoneal injection of pentobarbital sodium chloride (0.1 mL/100 g of body weight; Ceva santé animal, Bordeaux, France). Each animal was maintained in observation for a few minutes for security reasons; then, cardiac exsanguination was performed. The blood was centrifuged and the serum was frozen at −80 °C. Femurs and tibias were dissected free of connective and fat tissues and stored at −20 and +4 °C, respectively.

### 4.2. SR Administration

SR (S 12911-2) was supplied by Servier Laboratories (Technologie Servier, Orléans, France). Carboxymethylcellulose 0.5% (CMC) and SR were mixed in order to obtain a final concentration of 83.4 mg/mL. Rats were force-fed by stomach tube once daily with SR + CMC 0.5% at 625 mg/kg or with CMC 0.5% as control, 5 days a week [1]. Dose quantity was fixed based on previous reports [1].

### 4.3. Exercise Protocol

Rats performed jumping exercises for 8 weeks. During the first week, the rats were familiarized with the impact EXE protocol: the initial height was 25 cm, and this was gradually increased to 45 cm. Rats were submitted to 10 drops per day, 5 days/week for 8 weeks. As previously described, the rats were lifted horizontally for the jump, thus causing them to land on all four feet [2].

### 4.4. Bone Densitometry Measurements

Bone mineral density (BMD) and bone mineral content (BMC) were measured by dual-energy X-ray absorptiometry (DXA) (Discovery, Hologic, Bedford, MA, USA) using a specific small animal body composition mode [3]. Both measurements were taken once at week 0 (W0), before surgical operation, and once at week 8 (W8), before sacrifice. The root mean square coefficients of variation (CVs) of whole-body BMC and BMD were 1.19% and 1.4%, respectively, and were determined from three repeated measures with repositioning on 30 animals [3].

### 4.5. Morphological Characteristic of Trabecular Bone

The trabecular microarchitecture of the distal metaphysis of the left femurs was studied post-mortem using high-resolution micro-computed tomography imaging (µCT) (Skyscan 1072; Skyscan, Kontich, Belgium as previously described [3]. Briefly, the X-ray source was set at 80 kV and 100 µA with an isometric pixel size 15.49 µm. For each sample, 225 slices were selected and analyzed by NRecon and CTan software (skyscan 1072, Skyscan, Belgium). The following parameters were measured: bone volume fraction BV/TV (%), trabecular thickness Tb.Th (mm), trabecular spaces Tb.Sp (mm), and trabecular number Tb.N (1/mm).

### 4.6. Morphological Characteristic of Cortical Bone

The cortical bone of all left femurs was analyzed with the same acquisition characteristics as trabecular bone described above. The cortical porosity Ct.Po (%), pore number Po.N (number per mm), and pore spacing Po.Sp (mm) were measured as previously described [4]. Briefly, cortical bone and trabecular bone were separated and then converted to a binary image using a threshold adapted to each sample to determine cortical bone area. After reconstruction, the cortical bones (porosity, thickness, marrow area, and total area) were analyze using the CT analyzer software (MATLAB version R2013b, MathWorks).

### 4.7. Bone Mechanical Testing

The mechanical properties of all left femurs were assessed by a three-point bending test with a universal testing machine (Instron 3343, Instron, Bayswater, Australia). Femurs were secured on the two lower supports separated by a distance of 20 mm with a loading rate of 1 mm/min. The following mechanical parameters were recorded: ultimate strength (N), yield point stress (N), moment of inertia (mm^4^), and stiffness (N/mm). This protocol was adapted from the method of Maurel et al. [3].

### 4.8. Biochemical Analysis

Bone turnover markers were analyzed at W0 and W8 (end of the protocol). The biomarkers analyzed were osteocalcin (OCN) and bone alkaline phosphatase (ALP) for bone formation and the N-terminal telopeptide of type I collagen (NTX) for bone resorption. They were assayed in duplicate by ELISA. ELISA kits were obtained from EIAab (Wuhan Eiaab Science Co., Ltd., Wuhan, Zhangjiakou, Hebei, China) and used according to the manufacturer’s instructions.

### 4.9. Bone Immunostaining

#### 4.9.1. Bone Preparation

Bone explants (tibias) were fixed in formalin 4% and cut transversally at the proximal metaphysis of the left tibia with a high-speed rotary tool (Dremel 300, Dremel, Racine, WI, USA) without embedding. This method was adapted from the protocol of Vatsa et al. [5] on mice and used previously by our lab [6]. Bone slices were decalcified in osteosoft overnight (Merck, Darmsatdt, Germany) and blocked in blocking buffer (Phosphate Buffered Saline (PBS) containing 5% horse serum, 5% glycine, and 0.1% Triton X-100) for 2 h.

#### 4.9.2. Cleaved Caspase-3 Staining

For cleaved caspase-3 staining, bone sections after the blocking step were incubated with a monoclonal cleaved caspase-3 antibody (Cell signaling Technology Inc., Danvers, MA, USA) (dilution 1:200) and then incubated with a secondary antibody Dylight^TM^ 488 anti-rabbit (Rockland Immunochemical, Pottstown, PA, USA) (dilution 1:400). Bone sections were washed and then stained with DAPI for nucleus detection and mounted in Vectashield^TM^ mounting media (Vector Laboratories, Burlingame, CA, USA).

#### 4.9.3. Nile Red Staining

Bone sections were stained with Nile red (Sigma-Aldrich, Saint-Quentin-Fallavier, Isere, France). The protocol used was adapted from Fowler SD et al. [7]. Briefly, a stock solution of Nile red (1 mg/mL) was prepared in acetone and protected from light. A working solution was freshly prepared by a mix of stock solution (50 µL) and glycerol 75% (10 mL). A drop of working solution was added to Vectashield^TM^ mounting media (Ve Vector Laboratories, Burlingame, CA, USA) before imaging.

#### 4.9.4. Image Acquisition

Epifluorescence images were obtained with a Microvision microscope BA400 associated with an AE21 camera (Motic Biological). The objective magnification was 40x. Images were analyzed with Image J software. The number of osteocytes was determined by DAPI, the number of apoptotic cells by cleaved caspase-3, and lipids cells by Nile red and expressed as stained cells per mm².

### 4.10. Statistical Analysis

All numerical variables were expressed as mean ± SEM. Normality of distribution was tested. The critical *p*-value for statistical significance was *p* ≤ 0.05. Statistical analysis was performed using GraphPad Prism software (GraphPad Software, San Diego, CA, USA, www.graphpad.com accessed on 28 February 2021).

The effect of ovariectomy was analyzed using a non-parametric Mann–Whitney test to compare the Sh and O groups.

The separate effects and combined effect of SR and EXE were analyzed using a 2-way repeated-measures ANOVA test. Significant interactions were analyzed using a Fisher’s Least Significant Difference (LSD) test (GraphPad Software, San Diego, CA, USA). When the Fisher LSD test was significant, the effects of SR and EXE were considered as synergistic and non-significant interactions were not considered different.

## Figures and Tables

**Table 1:** Densitometric characteristics and body composition: whole-body bone mineral content (BMC) and bone mineral density (BMD) increase (difference to W0), fat and lean mass, and weight (difference to W0). Rats in the O group were ovariectomized. Rats in OE and OESr were subjected to 10 impacts per day, 5 days a week for 8 weeks. Rats in Sr and OESr groups received 625 mg/kg/day of SR. The critical *p*-value was *p* ≤ 0.05; NS: non-significant; a: vs. Sh; b: vs. O; c: vs. OE; d: vs. OSr and e: vs. OESr.

Variables	Sh	O	OE	OSr	OESr
Whole body BMC (g)	3.47 ± 0,66 c d e	2.48 ± 0.63 c d e	4.16 ± 0.57 a b	4.22 ± 0.32 a b	4.49 ± 0.99 a b
Whole body BMD (g/cm^2^)	0.039 ± 0.009 b	0.029 ± 0.012 vs. all	0.043 ± 0.009 b	0.044 ± 0.016 b	0.047 ± 0.012 b
Weight (g)	78.89 ± 27.56 b c	114.63 ± 16.44 E e	101.55 ± 12.20 a e	94.38 ± 24.79 e	64.53 ± 6.95 vs. all
Lean Mass (g) (%of weight)	37.48 ± 19.57 46.91% vs. all	59.21 ± 17.23 51.65% a	52.13 ± 7.97 51.33% a	50.47 ± 18.97 53.48% a	43.65 ± 9.54 67.64% a
Fat Mass (g) (% of weight)	38.95 ± 11.47 48.75% b e	51.28 ± 11.48 44.74% a d e	44.90 ± 7.28 44.21% e	39.72 ± 10.86 42.09% b e	18.02 ± 7.06 27.92% vs. all

**Table 2:** Microarchitecture of trabecular and cortical bones at the left femurs. Rats in the O group were ovariectomized. Rats in OE and OESr were subjected to 10 impacts per day, 5 days a week for 8 weeks. Rats in Sr and OESr groups received 625 mg/kg/day of SR. The critical *p*-value was *p* ≤ 0.05; NS: non-significant; a: vs. Sh; b: vs. O; c: vs. OE; d: vs. OSr and e: vs. OESr.

Zones	Variables	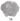	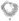	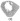	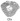	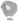
**Trabecular Bone**	BV/TV (%)	22.002 ± 1.647 b c e	6;287 ± 0.866 vs. all	19.017 ± 3.074 vs. all	22.227 ± 0.631 b c e	26.675 ± 0.534 vs. all
	Tb.Th (mm)	0.103 ± 0;115 vs. all	0.092 ± 0.004 vs. all	0.124 ± 0.008 vs. all	0.141 ± 0.006 vs. all	0.154 ± 0.009 vs. all
	Tb.N (1/mm)	2.143 ± 0.115 vs. all	0.683 ± 0.099 vs. all	1.522 ± 0.195 a b e	1.577 ± 0.083 a b e	1.739 ± 0.092 vs. all
**Cortical Bone**	Ct.Po(%)	0.038 ± 0.015 e	0.026 ± 0.012 e	0.031 ± 0.017 e	0.026 ± 0.016 e	0.010 ± 0.007 vs. all
	Ct.Th (mm)	0.475 ± 0.038 vs. all	0.413 ± 0.026 a d e	0.398 ± 0.032 a	0.503 ± 0.044 a b	0.512 ± 0.035 a b
	Po.N (1/mm)	0.011 ± 0.004 b d e	0.007 ± 0.003 a e	0.008 ± 0.004 a e	0.006 ± 0.003 a e	0.003 ± 0.002 Vs.

**Table 3:** Biomechanical testing of left femurs. Rats in the O group were ovariectomized. Rats in OE and OESr were subjected to 10 impacts per day, 5 days a week for 8 weeks. Rats in Sr and OESr groups received 625 mg/kg/day of SR. The critical *p*-value was *p* ≤ 0.05; NS: non-significant; a: vs. Sh; b: vs. O; c: vs. OE; d: vs. OSr and e: vs. OESr.

Variables	Sh	O	OE	OSr	OESr
Ultimate strength (N)	134.193 ± 11.313 d e	144.433 ± 11.040 e	145.163 ± 13.110 e	153.405 ± 11.234 a	157.778 ± 13.046 a b c
Cross-sectional area (mm^2^)	3.944 ± 0.258 vs. all	4.556 ± 0.176 vs. all	4.996 ± 0.0440 a b	4.941 ± 0.368 a b	5.274 ± 0.528 a b
Yield point stress (N)	269.313 ± 34.599 c e	256.185 ± 20.202 NS	232.273 ± 18.045 a d	264.452 ± 21.385 c	243.035 ± 34.0,857 a b
Moment of inertia (mm^4^)	4.207 ± 4.459 c e	4.818 ± 0.435 c e	5.555 ± 0.751 a b	4.872 ± 0.585 ns	5.776 ± 0.857 a b
Stiffness (N/mm)	221.410 ± 25.469 d e	223.143 ± 25.247 d e	227.152 ± 25.271 d e	241.152 ± 25.082 vs. all	256.580 ± 25.469 vs. all

**Table 4:** Bone remodeling markers of Wistar female rats. Bone alkaline phosphatase (ALP) and osteocalcin (OCN) were bone formation markers and N-telopeptide (NTX) a bone resorption marker. Rats in the O group were ovariectomized. Rats in OE and OESr were subjected to 10 impacts per day, 5 days a week for 8 weeks. Rats in Sr and OESr groups received 625 mg/kg/day of SR. The critical *p*-value was *p* ≤ 0.05; NS: non-significant; a: vs. Sh; b: vs. O; c: vs. OE; d: vs. OSr and e: vs. OESr.

Variables	Sh	O	OD	OSr	OESr
ALP (U/L)	1.686 ± 0.694 d e	2.314 ± 0.844 d e	1.808 ± 0.465 d e	4.189 ± 0.839 vs. all	3.110 ± 0.731 vs. all
OCN (pg/ml)	1.577 ± 0.364 b e	1.222 ± 0.346 vs. all	1.538 ± 0.622 b e	1.658 ± 0.466 b e	1.879 ± 0.698 vs. all
NTX (nm)	0.781 ± 0.152 d e	0.857 ± 0.076 d e	0.745 ± 0.103 d e	0.585 ± 0.086 a b c	0.622 ± 0.083 a b c

## Data Availability

Not applicable.
